# Dynamic Changes in Myocardial Function Early and Long-Term After Mitral Valve Surgery Detected by Speckle-Tracking Echocardiography

**DOI:** 10.3390/jcm15155761

**Published:** 2026-07-23

**Authors:** Maria Concetta Pastore, Alfonso Santoro, Elena Placuzzi, Maria Alma Iuliano, Francesca Rubina Ginetti, Francesca Vannuccini, Federica Marrese, Lorenzo Tanzi, Gianfranco Montesi, Sandro Sponga, Martina Rizzo, Giulia Elena Mandoli, Luna Cavigli, Marta Focardi, Flavio D’Ascenzi, Serafina Valente, Roberto Pedrinelli, Matteo Cameli

**Affiliations:** 1 Department of Medical Biotechnologies, Division of Cardiology, University of Siena, 53100 Siena, Italy; mariaconce.pastore@unisi.it (M.C.P.); matteo.cameli@unisi.it (M.C.); 2 Cardiac Surgery Unit, Cardiothoracic and Vascular Disease Department, Santa Maria Alle Scotte Hospital, University of Siena, 53100 Siena, Italy; 3 Cardiac, Thoracic and Vascular Department, University of Pisa, 56126 Pisa, Italy

**Keywords:** mitral regurgitation, cardiac surgery, speckle tracking, myocardial work, echocardiography

## Abstract

**Background:** It is known that mitral valve surgery produces an early reduction in hemodynamic overload after the procedure. However, the reduction in left ventricular (LV) ejection fraction (EF) may persist over the long term. Speckle-tracking echocardiography (STE) provides more sensitive indices of cardiac performance in MR. Myocardial work (MW) evaluates LV performance by considering the effect of LV afterload. The aim of this study was to describe the acute and late postoperative changes in cardiac function and hemodynamics using STE and MW indices in patients with primary MR and to find echocardiographic predictors of postoperative symptom improvement. **Methods:** Consecutive patients with severe MR and preserved LV EF undergoing mitral valve repair or replacement were prospectively enrolled. Exclusion criteria were atrial fibrillation, previous cardiac surgery, and concomitant aortic or coronary surgery. Patients underwent clinical, biohumoral and echocardiographic evaluation before surgery and within one week after surgery during the hospitalization period. Then, 32 of them underwent a new echocardiographic exam, completed by MW, at long-term follow-up. **Results:** Overall, 42 patients were enrolled (mean age 65 ± 12 years, 50% male, mean LV EF 59 ± 4%). After intervention, 48% of patients showed a reduction in LV EF (n = 20), while most patients showed reductions in STE and MW parameters across all chambers. Particularly, the global work index (GWI) was reduced in 79% of patients (n = 33). After a mean long-term follow-up of 26 ± 5 months, all STE parameters significantly improved in >70% of patients (n = 22), while LV EF maintained its initial reduction. A baseline value of LV GLS > −17.7% identified patients with persistence of symptoms at long-term follow-up (AUC 0.68, *p* = 0.02). **Conclusions:** After an initial decline, LV function detected by GLS improved over the long term after mitral valve surgery, paralleling a reduction in symptoms. STE indices might provide additional value for the long-term follow-up of patients undergoing mitral valve repair/replacement.

## 1. Introduction

Primary mitral regurgitation (MR) is the first-most common valvular disease and is caused by damage to the intrinsic anatomic structures of the mitral valve (leaflet, chordae, papillary muscles, and annulus). It is characterized by a high burden in terms of quality of life, morbidity, and mortality [1,2].

Surgical treatment (mitral valve repair or replacement) is considered the best therapeutic strategy for patients with severe symptomatic MR. However, MR surgery can lead to an early reduction in hemodynamic overload after the procedure and also to a slight reduction in left ventricular (LV) ejection fraction (EF), even in patients with normal baseline values, which can persist during long-term follow-up [3,4]. Speckle-tracking echocardiography (STE) has been shown to provide more sensitive indices of the performance of all cardiac chambers in different clinical settings, including MR and heart failure [5,6]. In both primary and secondary MR, its prognostic relevance has been analyzed in different studies, suggesting its superiority over LV EF in assessing remodeling changes and LV dysfunction related to primary valvular disease [7]. Particularly, impaired basal global longitudinal strain (GLS) was associated with postoperative LV EF and subsequently with poor outcomes and higher mortality, suggesting that this parameter is more sensitive in detecting subclinical dysfunction in asymptomatic patients and thus provides a useful parameter to optimize surgical timing [8,9]. In addition, left atrial (LA) evaluation by STE has shown its utility in MR, since global peak atrial longitudinal strain (PALS) has emerged as an additional predictor of poor prognosis, clinical outcomes and functional status, as well as a marker of LA fibrosis [10].

However, STE is still dependent on loading conditions, which are particularly important in valve heart disease and can precipitate severe MR.

Myocardial work (MW) is a novel echocardiographic method that non-invasively estimates LV performance by integrating strain data with LV pressure, estimated from peripheral arterial blood pressure, into pressure–strain loops, thereby accounting for the effects of afterload. MW indices have been shown to correlate with myocardial oxygen consumption, overcoming some of the limitations of strain-only techniques by providing more physiologically accurate, load-independent parameters [11].

MW has already shown its reliability and accuracy in different clinical settings, including valvular disease and heart failure, correlating accurately with invasive measurements of LV filling pressures [11,12,13,14].

The variations in MW parameters and their prognostic value have been studied in patients with various grades of secondary MR undergoing transcatheter edge-to-edge repair [15,16]. However, neither the link between MW assessed by STE and the hemodynamic changes occurring during the acute postoperative phase in patients with primary MR, nor its relationship with functional class and symptoms during long-term follow-up, has been investigated.

The aim of this study was to describe the acute and late postoperative changes in cardiac function and hemodynamics using basic echocardiography, STE and MW indices in patients with primary MR and to find preoperative predictors of improvement in NYHA class at long-term follow-up.

## 2. Methods

### 2.1. Study Population

In this observational prospective study, patients affected by severe primary MR, referred for surgical treatment (mitral valve repair or replacement) were enrolled. Before the intervention, a comprehensive evaluation was made, including clinical characteristics and laboratory exams. At the time of recruitment, physical examination and electrocardiography (ECG) were performed, and blood pressure was measured within ten minutes before the echocardiographic exam. The severity grade of valvular disease was evaluated according to international guidelines [17].Patients with reduced LV EF (<50%), previous cardiac surgery, the concomitant presence of other valvular disease > moderate severity or coronary artery disease with indications for surgery, a poor acoustic window or atrial fibrillation at the time of the exam were excluded.

After the intervention, patients underwent an additional examination (within one week), including clinical, laboratory and echocardiographic exams, completed by STE and MW.

**Figure 1:**
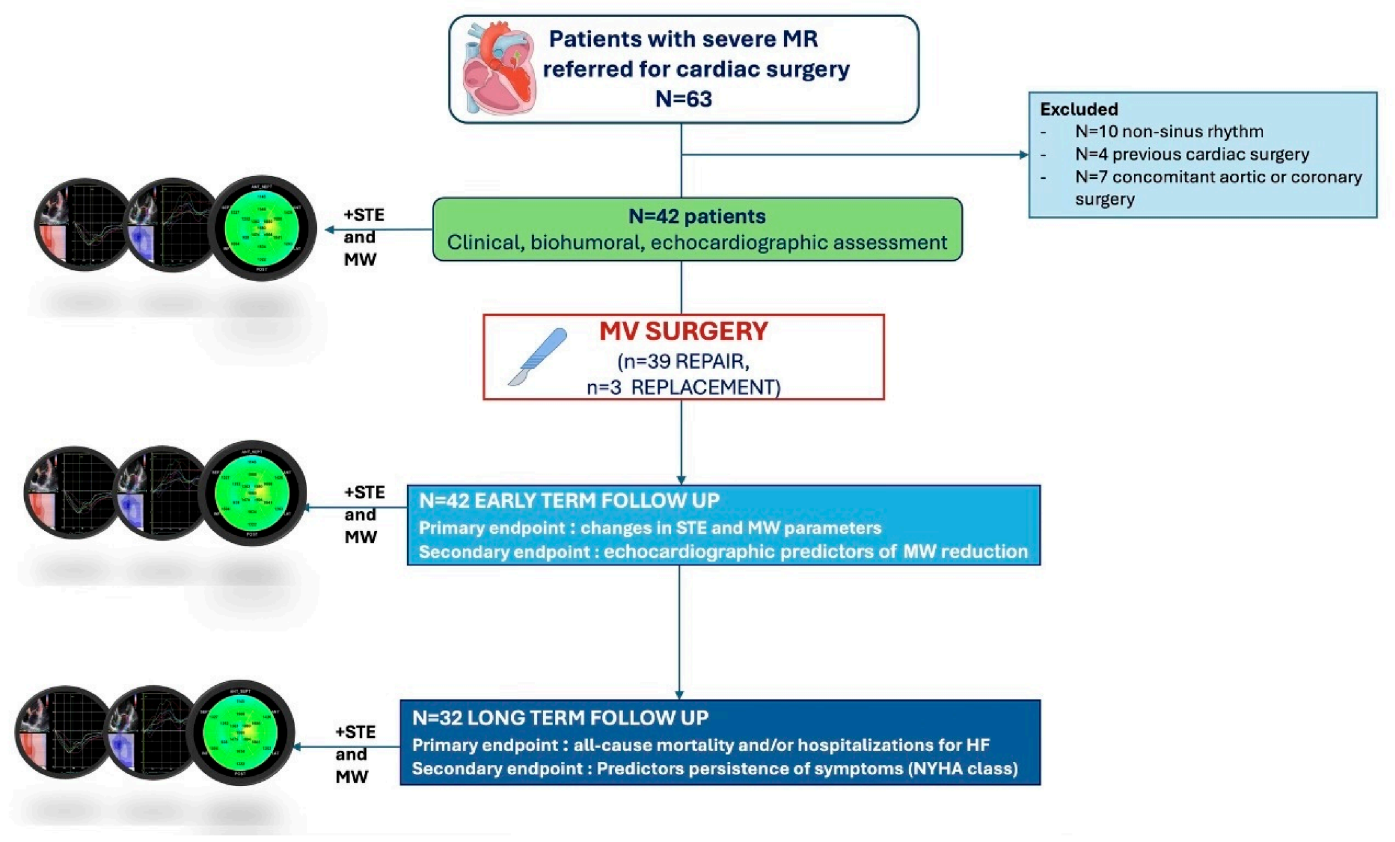
Algorithm of enrollment. MR, mitral regurgitation; MW, myocardial work; STE, speckle-tracking echocardiography.

Moreover, at least six months after the intervention, a subgroup of patients repeated clinical, laboratory and echocardiographic exams to assess long-term follow-up (Figure 1).

**Figure 2:**
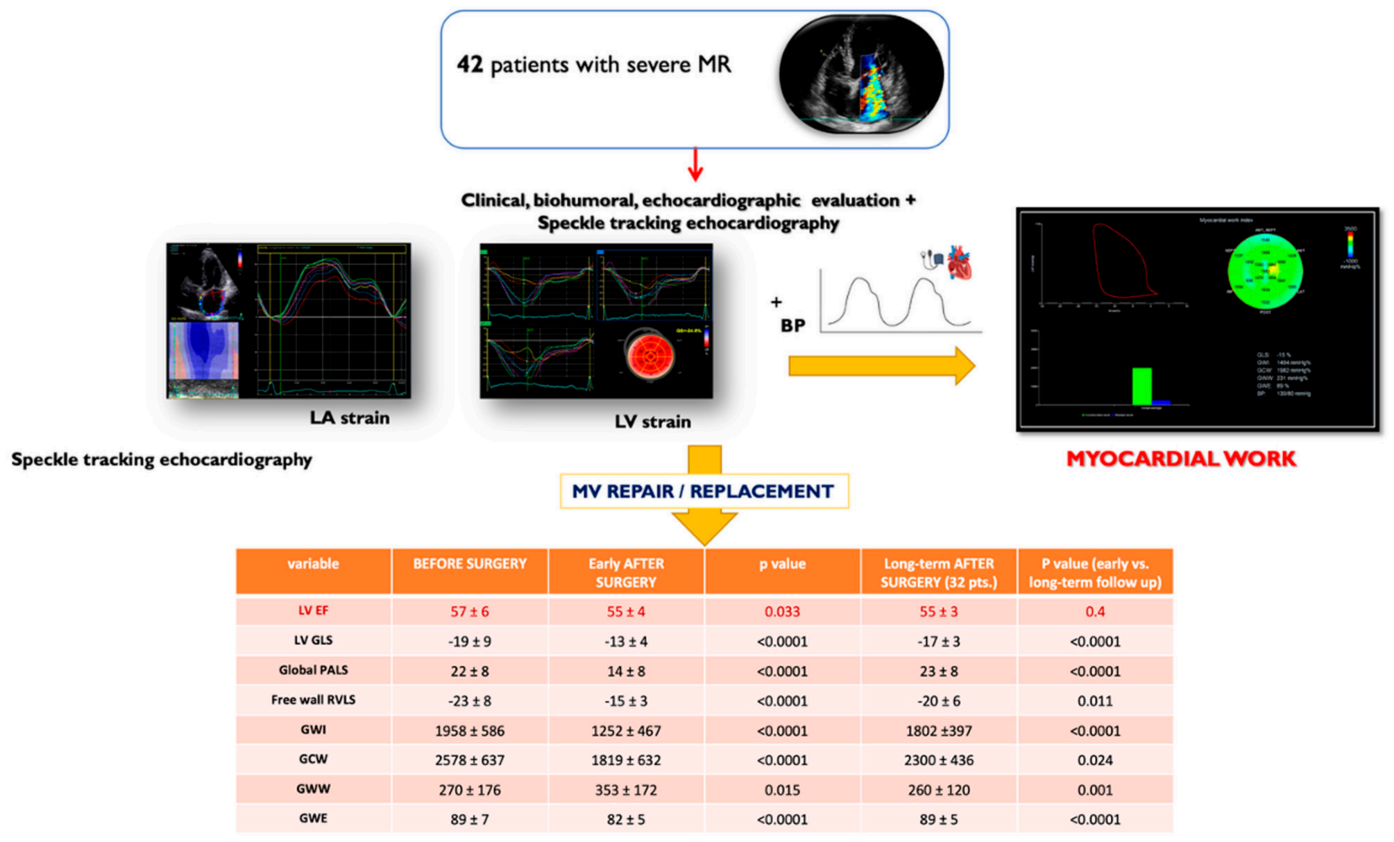
Central illustration. Study design and main results. GCW, global constructive work; GLS, global longitudinal strain; GWE, global work efficiency, GWI, global work index, GWW, global wasted work; LV, left ventricle; PALS, peak atrial longitudinal strain; RVLS, right ventricular longitudinal strain.

The primary endpoint was the changes in STE and MW parameters early after surgery and at long-term follow-up. Secondary endpoints were the identification of echocardiographic predictors of MW reduction in the early postoperative phase and the persistence of symptoms (assessed by New York Heart Association, NYHA class) at long-term follow-up (Figure 2 central illustration).

All patients provided written informed consent to participate in the study, and all study procedures were performed in accordance with the Declaration of Helsinki.

### 2.2. Basic Echocardiography Measurements

2D echocardiographic assessment was performed according to the recommendations of the American Society of Echocardiography (ASE) and the European Association of Cardiovascular Imaging (EACVI) [18,19], using a high-quality ultrasound system (Vivid E9; GE, Milwaukee, WI, USA) and a 2.5 MHz transducer with the patient in the left lateral decubitus position. Ultrasound settings were manually adjusted to optimize grayscale definition of the endocardium based on patient characteristics.

Regarding the preoperative echocardiographic parameters, LV diameters and thickness were measured from the parasternal long-axis view, and left intracavitary volumes were measured from the apical four-chamber view. Subsequently, LV EF was calculated using the modified Simpson biplane method.

Right ventricular diameters were evaluated in the apical four-chamber view.

LV and LA volumes were measured using the biplane disk sum method (modified Simpson formula) at end-diastole from the apical four-chamber window and were then indexed according to the patients’ body surface area.

Maximum early diastolic (E) and late diastolic (A) velocities were assessed by transmitral pulsed-wave Doppler to calculate the E/A ratio. Peak systolic (S′), early diastolic (e′), and late diastolic (a′) annular velocities were obtained by tissue Doppler imaging (TDI) at the septal and lateral mitral annulus, from which e′avg and the E/e′avg ratio were calculated; tricuspid annular S′ velocity was additionally obtained for right ventricular functional assessment. However, the postoperative E/E’avg reliability as an index of LV filling pressures is less reliable due to mitral annuloplasty rings and prosthetic valve implantation, which alter mitral geometry and may overestimate filling pressures.

Tricuspid annulus plane systolic excursion (TAPSE) was measured using the M-mode function in the apical four-chamber view. Right ventricular dimensions and function were measured in the apical four-chamber view. Systolic pulmonary artery pressure (sPAP) was estimated as the sum of the peak trans-tricuspid systolic pressure gradient and right atrial pressure derived from the diameter and collapsibility of the inferior vena cava.

MR severity was assessed with color-flow Doppler using a four-grade scale (mild, moderate, medium, and severe regurgitation). The possible presence of valve flail or leaflet prolapse was then assessed. The mitral annulus diameter was measured at end-systole from the apical two-, three-, and four-chamber views, and the annulus was considered dilated when >35 mm, as recommended by the ASE/EACVI [19,20] guidelines. Using color-flow Doppler, the vena contracta was measured in patients with a single regurgitant jet. Other valvular heart diseases were quantified by 2D echocardiography according to the EACVI recommendation [20].

### 2.3. Speckle Tracking and Myocardial Work Analysis

To measure LV GLS, apical four-, two- and three-chamber two-dimensional grayscale images with a stable electrocardiographic recording were analyzed. Atrial strain was calculated using the apical four- and two-chamber views, using the QRS wave as the starting point of the cardiac cycle. Dedicated views were utilized for LV, LA and right ventricular analysis, allowing more reliable delineation of the atrial endocardial border. Three consecutive heart cycles were recorded and averaged. The frame rate was 60–80 frames/s. Analysis was performed offline by a single experienced and independent echocardiographer using a commercially available semiautomated two-dimensional strain software (EchoPac, GE, Milwaukee, WI, USA) with automated functional imaging (AFI) algorithms dedicated to LV and LA. LV GLS was calculated as the average of the longitudinal strain curves obtained from the four-, two- and three-chamber views. Global PALS was calculated at the end of the reservoir phase as the average of the values observed in all LA segments in the four- and two-chamber views. Right ventricular free-wall longitudinal strain (RVFWLS) was derived from a region of interest comprising three segments (basal, mid, and apical) of the RV free wall.

For subsequent MW analysis, the operator confirmed the timing of valvular events using the apical three-chamber view synchronized with the ECG trace. Brachial arterial pressure measured during the echocardiographic exam (in the supine position, both preoperatively and postoperatively) was used by the software to fit a non-invasively estimated LV pressure curve [21,22], based on peak arterial pressure and valve events. The software automatically created a pressure–deformation loop derived from the estimated LV pressure curve and GLS and generated four myocardial performance indices: global work index (GWI), which is the total work performed by the left ventricle from mitral valve closure to mitral valve opening (encompassing isovolumic contraction, ejection, and isovolumic relaxation); global constructive work (GCW), which is the positive work done during shortening in systole plus the negative work done during lengthening period during isovolumetric relaxation; global wasted work (GWW), which is the negative work performed during lengthening in systole plus the work performed during shortening in isovolumetric relaxation; and global work efficiency (GWE), which is calculated as constructive work divided by the sum of constructive work and wasted work [23,24]. Images for postoperative MW analysis were acquired when patients were considered hemodynamically stable, had a normal heart rate, were in the cardiac surgery ward, and were not receiving vasoactive or inotropic therapy. Inter-operator and intra-operator reproducibility were blindly tested in a random sample of 10 patients.

### 2.4. Statistical Analysis

Continuous variables were expressed as the mean ± SD (standard deviation), and binary variables were expressed as counts or percentages. Continuous variables were tested for normality using the Kolmogorov–Smirnov test and were then compared using the *t*-test. Categorical variables were compared using the χ^2^ test. For the primary endpoint, changes over time in the echocardiographic variables were tested using the paired-samples *t*-test.

Patients were first studied as a whole cohort and were then divided into subgroups based on the absence of symptoms, defined as NYHA class = 1, or the persistence of symptoms, defined as NYHA class > 1 at long-term follow-up. The groups were compared using the independent-samples *t*-test for the secondary endpoint. Receiving Operating Curves (ROC) were used to calculate the predictive value of advanced echocardiographic variables for the reduction in myocardial work parameters during the early postoperative phase and for the prediction of NYHA = 1 at long-term follow-up, including the area under curve (AUC), and sensitivity and specificity using standard definitions.

The variables selected using this process were discretized using the Youden index on the respective ROCs to find the threshold.

Univariate and multivariate logistic regression analyses were performed for exploratory purposes. Variables were selected for inclusion in the multivariate model based on their statistical significance in univariate analysis (*t*-test) and on biological plausibility.

Statistical Package for the Social Sciences (SPSS) version n.27 was used for the analyses. A *p* value lower than 0.05 was considered significant.

## 3. Results

### 3.1. General Population Characteristics

Overall, 42 patients were enrolled in this study. Almost half were male (48%), with mean age of 63 ± 15 years and preserved LV EF (57 ± 6%, Table 1). The most common risk factors were hypertension (61.9%), dyslipidemia (40.47%), and diabetes (7.14%). More than 20% had a history of heart failure, and 14% suffered from paroxysmal atrial fibrillation. Almost all patients were treated with mitral valve repair with annuloplasty (n = 39), including one with concomitant tricuspid valve repair, whereas only three with mitral valve replacement with biological prostheses. The mean cross-clamp time was 78 ± 29 min, and the mean cardiopulmonary bypass time was 111 ± 30 min. 

### 3.2. Echocardiographic Parameters

Concerning the early postoperative phase (Table 2), all patients presented preserved LV EF, although an early reduction in systolic function was observed post-intervention (58 ± 4 vs. 55 ± 4% *p* = 0.03). Moreover, a reduction in right ventricular function indexes, including TAPSE (24 ± 4 vs. 16 ± 3 mm) and tricuspid s’ (0.15 ± 0.02 vs. 0.14 ± 0.17, *p* < 0.015) was detected. Seven patients showed mild residual mitral regurgitation.

LV end-diastolic volume (LVEDV) showed a postoperative reduction compared to baseline values (LVEDV: 130 ± 38 mL vs. 95 ± 31 mL; *p* = 0.03).

A decrease in LA volume was also observed early after surgery, as demonstrated by the reduction in LAVi (55 ± 20 mL/m^2^ vs. 46 ± 14 mL/m^2^; *p* < 0.0001). However, during long-term follow-up, LAVi increased slightly, although it remained significantly lower than the baseline measurement (LAVi: 50 ± 5 mL/m^2^; *p* < 0.001).

On the other hand, E/E’ appeared higher in the postoperative phase (E/e′: 11 ± 4 vs. 15 ± 4; *p* < 0.0001) and remained stable during long-term follow-up (E/e′: 15 ± 6; *p* < 0.2). However, this assessment may be less reliable due to the potential impact of the mitral annuloplasty ring and prosthetic valve implantation, which alter mitral geometry and may confound the accurate estimation of filling pressures.

### 3.3. STE and Myocardial Work Parameters

Early after the intervention (<1 week), only 48% of patients showed a reduction in LV EF (n = 20), while most patients showed a reduction in STE and MW parameters in all chambers (Figure 2. Central illustration). Particularly, the global work index (GWI) was reduced in 79% of patients (n = 33), with mean values decreasing from 1958 ± 586 to 1252 ± 430 mmHg%. The same was observed for global constructive work (GCW) (2578 ± 612 vs. 1819 ± 490 mmHg%) and global work efficiency (GWE) (89 ± 5% vs. 82 ± 5%), while global wasted work (GWW) increased (270 ± 178 vs. 353 ± 137 mmHg%).

In univariate and multivariate analyses including LV EF, LAVI, tricuspid s’ velocity by tissue Doppler imaging, global PALS, and right ventricular free-wall longitudinal strain, global PALS emerged as the only independent predictor of GWI reduction after surgery (odds ratio, OR = 1.26 [1.03–1.55]; *p* = 0.021).

In the sub-cohort of patients evaluated after a long-term follow-up of 26 ± 5 months (n = 32 patients; Figure 1), almost all STE parameters and MW indices increased, while LV EF did not show any relevant changes compared with the acute phase (55 ± 4 vs. 55 ± 3%; *p* value: 0.4) (Table 3).

GWI increased in almost 70% of the population (n = 22) at long-term follow-up, with a mean value of 1802 ± 397 mmHg%, compared with early post-intervention phase (1252 ± 467, *p* value < 0.0001). The other MW parameters also showed recovery from the post-acute phase of analysis, with GCW and GWE within the normality range, i.e., 2330 ± 496 mmHg% (*p* value 0.024) and 89 ± 5% (*p* value < 0.0001), respectively. In contrast, GWW decreased (260 ± 120 mmHg%, *p* value 0.001), showing similar values to the baseline evaluation (270 ± 176 mmHg%).

**Figure 3:**
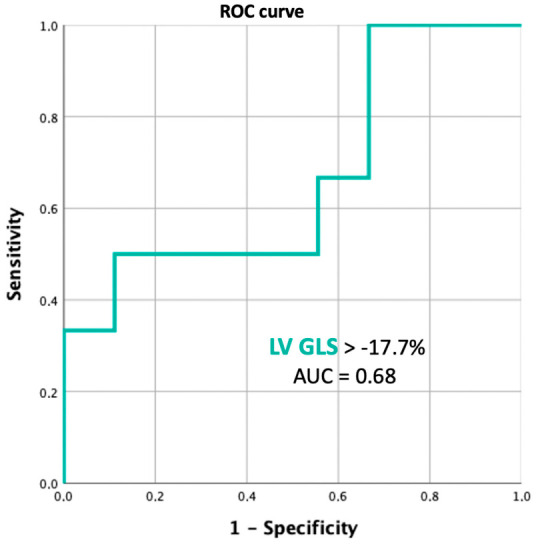
Receiver operating characteristic (ROC) curve for the prediction of persistence of symptoms at follow. GLS, global longitudinal strain; LV, left ventricle.

At baseline, nine patients had NYHA = 1, seventeen patients had NYHA = 2, and six patients had NYHA = 3. Overall, 30% of patients (seven of the twenty-three patients who had symptoms at baseline) showed persistence of symptoms (i.e., NYHA class > 1; particularly, four patients with NYHA = 2 and three patients with NYHA = 3) at long-term follow-up. Among the baseline clinical and echocardiographic parameters (Table 3), only the mitral E/A and LV GLS showed significant differences between patients with absence or persistence of symptoms at follow-up (NYHA class = 1 or >1). ROC analysis was performed as an exploratory analysis, considering the small number of events (n = 7). LV GLS > −17.7% showed a modest but significant predictive value for the persistence of symptoms at long-term follow-up (AUC 0.68, *p* = 0.02, Figure 3), while E/A did not (AUC 0.53, *p* = ns).

Overall reproducibility of the STE parameters was excellent for the left heart (ICC: 0.95 [0.95–0.96] for LV GLS and all MW parameters and 0.93 [0.90–0.95] for global PALS) and good for RV strain (ICC = 0.88 [0.86–0.89]).

## 4. Discussion

The present study is the first to investigate early and late postoperative changes in MW assessed by STE parameters in patients undergoing surgery for severe MR. Although this should be considered a pilot study due to the small number of patients, our findings suggest that STE indices are more sensitive in describing the initial decline, followed by long-term improvement, in LV function after mitral valve surgery, offering a more accurate and relatively load-independent assessment of LV performance after surgery in patients with primary MR. Moreover, the exploratory analysis showed a potential association between global PALS and early postoperative MW decline, as well as between LV GLS and long-term symptoms after surgery.

Severe primary MR induces a hemodynamic condition characterized by chronic volume overload of the left atrium, leading to an increase in LV preload. The prolonged maintenance of this state can trigger structural remodeling with dilation of the affected chamber.

Persistent hemodynamic alterations may result in increased systolic wall stress and afterload, promoting progression from a compensated state to an asymptomatic state of decompensation. This can occur even in the presence of preserved LV EF, which does not accurately reflect the extent of LV contractile impairment. Moreover, LV remodeling cannot be fully represented by a sole increase in end-systolic diameter; apical remodeling should also be considered to achieve a comprehensive morphological assessment [25,26,27].

Previous studies have highlighted the superior sensitivity of LV GLS in detecting subclinical dysfunction compared to LV EF. A meta-analysis by Ueyama et al., involving 5267 patients undergoing mitral valve intervention, demonstrated that lower baseline GLS values (ranging between −17.9% and −20.5%) were consistently associated with postoperative LV EF reduction and adverse cardiac remodeling [7]. Furthermore, impaired baseline GLS emerged as an independent predictor of all-cause mortality and major adverse cardiac events. Another meta-analysis of 2358 patients with severe primary MR and preserved LV EF confirmed the predictive value of LV GLS for both postoperative LV EF decline and long-term survival, particularly in asymptomatic individuals in whom early myocardial dysfunction may otherwise go undetected [9].

LV GLS appears to be more effective in predicting persistent postoperative reductions in LV function, as pre-existing contractile dysfunction may be masked by the reduced LV afterload, allowing temporary preservation of an apparently normal EF. However, after surgical correction, this condition may lead to a clinically significant and persistent decline in LV EF.

These observations are reinforced by Pandis et al., in which preoperative GLS values were found to predict a significant reduction in LV EF, both in absolute terms and relative to baseline values, in patients with severe chronic primary MR undergoing mitral valve repair. The authors hypothesized that an excessive preoperative improvement in longitudinal GLS may represent a maladaptive hyper-compensatory response of the longitudinal myocardial fibers to elevated preload and may anticipate postoperative deterioration in LV function [28]. If confirmed in further studies, this feature could prove highly useful in optimizing the timing of surgical intervention in patients with primary MR, both symptomatic and, especially, asymptomatic patients [5].

Our finding that a baseline GLS value > −17.7% may predict persistent symptoms during after-surgery follow-up is supported by a recent study involving 103 patients, in which LV GLS emerged as an independent predictor of LV reverse remodeling, with a threshold value of −16.25 (closely matching our results) [29].

In patients with severe MR, LV GLS and GWI have shown independent associations with cardiovascular events, as demonstrated in the study by Verbecke et al. involving patients with heart failure and secondary MR [30]. However, the clinical applicability of these cut-off values for surgical referral remains limited, largely due to the different pathophysiological profiles of primary and secondary MR [31].

However, GLS has emerged as a reliable tool for optimizing surgical timing in MR, particularly in asymptomatic patients with subclinical myocardial dysfunction. Supporting this, Hiemstra et al. reported that a GLS value > −20.6% was associated with worse long-term outcomes in a cohort of 593 patients with severe primary MR undergoing surgery, reinforcing the role of GLS in early risk stratification, even before traditional guideline thresholds are met [32].

It is important to highlight that PALS also represents a reliable prognostic parameter for MR, as it correlates with both myocardial damage and clinical presentation in patients with MR [10,33]. This parameter has already demonstrated its usefulness in various clinical settings, such as heart failure and atrial fibrillation [34], and serves as an additional tool in the assessment of diastolic function [35,36]. Furthermore, PALS has proven to be more sensitive than LV GLS in detecting myocardial damage or dysfunction in patients with severe and moderate MR and preserved EF [33].

In fact, the assessment of PALS enables estimation of LV filling pressures and early identification of LA dysfunction, which is associated with increased afterload due to the chronic volume overload typical of severe MR [37,38]. Alterations in PALS, which can be linked to early atrial remodeling, have also been shown to correlate with functional class and symptoms, confirming previous findings [10,34], which suggest its usefulness in the prognostic evaluation of both symptomatic and asymptomatic patients with MR.

In the present study, among all the echocardiographic parameters analyzed, only PALS showed a significant association with postoperative reduction in GWI. This may be explained by the higher sensitivity of PALS in the early detection of ultrastructural and biochemical alterations affecting the left atrium, which are associated with changes in cardiac function even during asymptomatic stages of valvular disease.

The correlation between PALS and the postoperative reduction in GWI, consistent with the decline observed in other MW indices, may be due to remodeling processes and myocardial alterations resulting from the hemodynamic overload induced by MR. These findings support the role of PALS as a useful parameter for identifying the early stages of the disease.

The evaluation of valvular heart disease using MW parameters seems to offer a more accurate evaluation of myocardial function, as it is less affected by loading conditions, namely, changes in preload and afterload. This is particularly relevant in the context of clinically significant valvular heart disease, where MW parameters have already proven their validity, as well as their association with ventricular remodeling and maladaptive responses [39]. In another study conducted in asymptomatic patients with moderate-to-severe aortic stenosis and preserved LV EF, GWI and GCW were inversely correlated with the severity of stenosis [40]. Threshold values of <1951 mmHg% for GWI and <2475 mmHg% for GCW were associated with a worse prognosis.

In aortic regurgitation, Meucci et al. also demonstrated a correlation between GWI and GCW values and disease severity. Moreover, a marked postoperative reduction in GWI was associated with a lower degree of reverse remodeling and with structural changes attributable to underlying myocardial fibrosis [41].

Changes in MW parameters have also been observed and studied in secondary MR, where reduced energetic efficiency, reflected by increased GWW and GWE, was likely related to ventricular unloading into a low-pressure chamber. Conversely, GCW and GWI were found to be more severely impaired [14]. In our analysis, the changes observed in MW parameters are similar to those reported by Galli et al. in a study conducted in patients with severe primary MR undergoing transcatheter edge-to-edge valve repair [15]. In that study, echocardiographic evaluation was performed preoperatively and immediately prior to hospital discharge, followed by long-term follow-up. In the early postoperative period, an acute reduction was observed in LV EF, GLS, GWI and GCW, accompanied by an increase in GWW. At one year, an overall improvement in MW parameters was noted, although GWW remained impaired. Notably, the preoperative GWW value was identified as the only independent predictor of LV reverse remodeling.

In our study, a postoperative reduction in LV EF was observed in 50% of patients, accompanied by a generalized decline in all MW and STE parameters. Notably, GWI was reduced in nearly 80% of the analyzed population, suggesting that these parameters may be more sensitive in detecting subtle impairments in cardiac function and performance. The maintenance of normal or mildly elevated MW and GLS values in the preoperative phase may reflect an overall adaptive mechanism of the LV in response to increased preload due to the mitral valve defect. This condition leads to elevated wall stress and volumetric remodeling. After surgical correction of MR, in patients with postoperative LV EF reduction, underlying contractile dysfunction appears to persist—as indicated by the decline in MW parameters—despite relatively preserved LV EF. These findings confirm the higher accuracy and sensitivity of advanced echocardiographic indices in assessing cardiac function.

At long-term follow-up, LV EF remained stable compared with the early postoperative values. Approximately 70% of patients showed improvement in MW parameters, particularly GWI and GCW, which returned to normal values. GWE, a marker of myocardial work efficiency, also normalized. Furthermore, GWW, which reflects the amount of ineffective MW occurring during isovolumetric relaxation, showed a significant reduction, approaching—but not fully reaching—normal threshold values. This residual impairment likely reflects the presence of subclinical alterations, possibly related to myocardial fibrosis, that are not reversible even after surgical correction.

In the study by Nabeta et al., patients with severe MR undergoing surgical intervention and presenting with impaired preoperative LV GWI had worse clinical outcomes in terms of all-cause mortality, suggesting an additional prognostic value of this parameter. Although both LV GLS and GCW were also impaired, they did not reach statistical significance in predicting prognosis [42].

Therefore, these advanced echocardiographic indices may provide valuable additional information to optimize the timing of surgical intervention, particularly in patients with preserved LV systolic function and in asymptomatic individuals, with the aim of avoiding postoperative LV dysfunction and improving overall clinical outcomes. Moreover, if these results are confirmed in larger studies, MW may offer additional and more sensitive indices to detect subtle LV impairment and provide the right timing for intervention and stricter follow-up according to the patient’s phenotype.

Concerning the secondary endpoint, which should be considered as exploratory due to the low number of events, baseline LV GLS values seemed correlate significantly with the persistence of clinical symptoms despite mitral valve surgery. If these results are confirmed in larger studies, GLS may be considered as an additional parameter in the future to optimize the selection of patients with MR to refer for cardiac surgery, in order to identify patients most likely to benefit from surgery in terms of symptom improvement.

Taken together, although these results should be considered hypothesis-generating, LV GLS and MW may provide additional value in guiding a more intensive anti-remodeling therapeutic strategy, both in the immediate postoperative period and during subsequent follow-up, in patients with severe MR, thereby helping to optimize clinical outcomes after cardiac surgery, limit heart failure progression, and improve long-term quality of life.

## 5. Study Limitations

Despite the promising results and the potential for future developments arising from this research, some limitations should be acknowledged. First, the small sample size and the lack of a control group do not allow for generalizable conclusions and limit statistical power, requiring further evidence on the topic. Particularly, the prognostic regression analyses should be considered exploratory. Moreover, this was a single-center study with a maximum follow-up duration of three years (including incomplete long-term follow-up and potential selection bias), which is insufficient to assess long-term changes that might yield clinically relevant insights. Due to the limited sample size and incomplete medical records, per-group analysis according to the duration of mitral valve disease and type of surgical repair was not feasible, nor was the analysis of hard clinical endpoints.

Furthermore, despite the good results in terms of feasibility and reproducibility, the use of STE is still limited in patients with suboptimal acoustic windows and by the availability of the software. Moreover, the load dependence of STE measurement, the possible confounding effect of postoperative hemodynamic status, and the limited reliability of postoperative diastolic indices should be taken into consideration.

## 6. Conclusions

Cardiac surgery remains the first resolutive therapy for severe MR. However, it may affect cardiac function in the postoperative period due to both the surgical procedure and the hemodynamic changes resulting from the resolution of MR.

Parameters derived from STE and MW have proven to be more sensitive than LV EF in evaluating LV function both early and late after the surgical treatment of MR, as they are less affected by postoperative hemodynamic changes. These indices typically reveal a transient decline in function in the immediate postoperative period, followed by improvement during follow-up.

Notably, preoperative PALS was identified as a potential predictor of postoperative reductions in LV function, and GLS showed an association with the persistence of symptoms during long-term follow-up. These results suggest the need for new evidence to confirm these findings on a larger scale in order to consider the use of these parameters in the selection of candidates for MR surgery and the postoperative evaluation of patients undergoing surgical treatment for MR to guide medical therapy and follow-up planning.

## Figures and Tables

**Table 1:** Clinical, biohumoral and echocardiographic characteristics at baseline evaluation in the overall population.

Variables	Mean (Overall Population)	Standard Deviation (Overall Population)
AGE	63	15
BMI	26	5.5
SBP	125	19
DBP	75	11
HR	76	17
HB (g/dL)	14	1
LEUCOCYTES	11	1716
CREATININE (mg/dL)	1.01	0.27
TnHs (ng/L)	9.36	5.9
NA	141	2
K	4.2	0.4
IVS (mm)	11	2
E/A mitr	1.5	0.5
DT/_mitr	195	48
E/E’ avg	10	4
EDV IND (mL/m^2^)	68	21
ESV IND (mL/m^2^)	28	9
LV EF (%)	57	6
LAVI (mL/m^2^)	55	20
TDI_S_tric	0.14	0.03
RVEDD_avg_	30	3
TAPSE (mm)	23	4
RVFAC	46	9
sPAP (mmHg)	33	11
IVC (mm)	17	3
GLOBAL PALS	22	8
GLOBAL PACS	10	6
GLS	−18	9
RV free-wall longitudinal strain	−23	9
GWI (mmHg/%)	1743	586
GCW (mmHg/%)	2388	638
GWW (mmHg/%)	292	176
GWE (%)	86	7

BMI, body mass index; BSA, body surface area; DBP, diastolic blood pressure; DT, deceleration time; E/A, ratio of early (E) to late (A) transmitral flow velocities; E/E’avg, peak early diastolic “E” wave/medium early mitral annular velocity by tissue Doppler imaging; EDV IND, end-diastolic volume index; ESV, end-systolic volume index; HB, hemoglobin; HR heart rate; fwRVLS, free-wall right ventricular longitudinal strain; GLS, global longitudinal strain; GWI, global work index; GCW, global constructive work; GWW, global wasted work; GWE, global work efficiency IVS, interventricular septum; K, blood potassium; LV EF, left ventricle ejection fraction; LAVi, left atrium volume index; NA, blood sodium; PACS, peak atrial contraction strain; PALS, peak atrial longitudinal strain; RVEDD, right ventricular end-diastolic diameter RVFAC, right ventricular fractional area change; TAPSE, tricuspid annular plane systolic excursion; TDI-s, tissue Doppler imaging; SBP, systolic blood pressure. sPAP, systolic pulmonary artery pressure; TnHS, high-sensitivity troponin.

**Table 2:** Advanced echocardiographic characteristics before surgery, early after surgery and at long-term follow-up.

Variable	Before Surgery	Eary After Surgery (Within One Week)	*p* Value (Early After vs. Before Surgery)	Long-Term After Surgery (26 ± 5 Months)	*p* Value (Long-Term vs. Early After Surgery)
N° patients	42	42		32	
LV EF	57 ± 6	55 ± 4	0.033	55 ± 3	0.4
LV GLS	−19 ± 9	−13 ± 4	<0.0001	−17 ± 3	<0.0001
Global PALS	22 ± 8	14 ± 8	<0.0001	23 ± 8	<0.0001
RVFWLS	−23 ± 8	−15± 3	<0.0001	−20 ± 6	0.011
GWI	1958 ± 586	1252 ± 467	<0.0001	1802 ± 397	<0.0001
GCW	2578 ± 637	1819 ± 632	<0.0001	2300 ± 436	0.024
GWW	270 ± 176	353 ± 172	<0.015	260 ± 120	<0.001
GWE	89 ± 7	82 ± 5	<0.0001	89 ± 5	<0.0001
LVEDVi	68 ± 22	61 ± 4	0.031	52 ± 7	<0.0001
LVESVi	28 ± 9	26 ± 1	0.2	24 ± 5	0.019
LAVi	55 ± 20	46 ± 14	<0.0001	50 ± 5	0.001
E/e’avg	10 ± 4	15 ± 4	<0.0001	15 ± 6	0.2

LV EF, left ventricular ejection fraction; LV GLS, left ventricular global longitudinal strain; PALS, peak atrial longitudinal strain; RVFWLS, right ventricular free-wall longitudinal strain, E/E’avg, peak early diastolic “E” wave/mean early mitral annular velocity by tissue Doppler imaging; LVEDVi, left ventricular end-diastolic volume index; LVESVi, left ventricular end-systolic volume index; LAVi, left atrium volume index; GWI, global work index; GCW, global constructive work; GWW, global wasted work; GWE, global work efficiency.

**Table 3:** Baseline clinical, biohumoral and echocardiographic characteristics according to NYHA class at follow-up.

Variable	NYHA = 1	NYHA > 1	*p* Value
Age	60 ± 16	69 ± 10	0.13
BMI	25 ± 4	26 ± 7	0.69
BSA	1.8 ± 0.2	1.7 ± 0.2	0.31
sBP	128 ± 20	121 ± 18	0.34
dBP	77 ± 11	71 ± 9	0.18
HR	73 ± 14	75 ± 14	0.68
Hb (g/dL)	14 ± 1	13 ± 1.4	0.14
White blood cells (10^3^/μL)	6587 ± 1060	6177 ± 1160	0.37
Glucose (mg/dL)	94 ± 11	95 ± 13	0.9
Creatinine(mg/dL)	1.1 ± 0.3	1 ± 03	0.81
Tn-Hs (ng/L)	9.3 ± 6.5	10 ± 6	0.75
NA (mEq/L)	141 ± 1.5	141 ± 2	0.61
K (mEq/L)	4 ± 0.4	4 ± 0.3	0.98
IVS (mm)	11 ± 2	11 ± 2	0.34
PW (mm)	10 ± 1	10 ± 1	0.5
LV MASS INDEX	125 ± 30	116 ± 25	0.43
E/A mitr	1.1 ± 0.5	1.7 ± 0.5	0.02
DT/_mitr	197 ± 44	196 ± 32	0.95
E/E’ avg	11 ± 5	9 ± 4	0.23
EDV IND (mL/m^2^)	66 ± 27	67 ± 19	0.64
ESV IND (mL/m^2^)	26 ± 9	28 ± 8	0.6
LV EF (%)	58 ± 3	56 ± 5	0.4
LAVI (mL/m^2^)	50 ± 27	59 ± 16	0.4
TDI_S_tric (m/s)	0.14 ± 0.03	0.15 ± 0.05	0.87
RVEDD_avg_	31 ± 3	31 ± 3	0.96
TAPSE (mm)	23 ± 4	24 ± 4	0.88
RVFAC	46 ± 8	48 ± 10	0.65
sPAP (mmHg)	33 ± 5	35 ± 5	0.64
IVC (mm)	16 ± 3	17 ± 3	0.36
GLOBAL PALS	22 ± 6	22 ± 8	0.63
GLOBAL PACS	11 ± 7	8 ± 5	0.62
GLS	−21 ± 4	−16 ± 2	0.02
RV free-wall longitudinal strain	−23 ± 8	−21 ± 9	0.47
RV global longitudinal strain	−20 ± 6	−19 ± 7	0.69
GWI (mmHg/%)	2008 ± 382	1557 ± 529	0.05
GCW (mmHg/%)	2439 ± 615	1859 ± 543	0.02
GWW (mmHg/%)	288 ± 155	339 ± 223	0.54
GWE (%)	88 ± 7	85 ± 7	0.74

BMI, body mass index; BSA, body surface area; DBP, diastolic blood pressure; DT, deceleration time; E/A, ratio of early (E) to late (A) transmitral flow velocities; E/E’avg, peak early diastolic “E” wave/medium early mitral annular velocity by tissue Doppler imaging; EDV IND, end-diastolic volume index; ESV, end-systolic volume index; fwRVLS, free-wall right ventricular longitudinal strain; GLS, global longitudinal strain; GWI, global work index; GCW, global constructive work; GWW, global wasted work; GWE, global work efficiency; HB, hemoglobin; HR, heart rate; IVS, interventricular septum; K, blood potassium; LAVi, left atrium volume index; LV EF, left ventricle ejection fraction; NA, blood sodium; PACS, peak atrial contraction strain; PALS, peak atrial longitudinal strain; PWT, posterior wall thickness; RVEDD, right ventricular end-diastolic diameter; RVFAC, right ventricular fractional area change; SBP, systolic blood pressure; sPAP, systolic pulmonary artery pressure; TAPSE, tricuspid annular plane systolic excursion; TDI-s, tissue Doppler imaging; TnHS, high-sensitivity troponin.

## Data Availability

The data presented in this study are available on request from the corresponding authors.

## Abbreviations

The following abbreviations are used in this manuscript:

ASE	American Society of Echocardiography
BMI	body mass index
BSA	body surface area
DBP	diastolic blood pressure
DT	deceleration time
E/A	ratio of early (E) to late (A) transmitral flow velocities
E/E’avg	peak early diastolic “E” wave/medium early mitral annular velocity by tissue Doppler imaging
EDV IND	end-diastolic volume index
EACVI	European Association of Cardiovascular Imaging
ESV	end-systolic volume index
HB	hemoglobin
HR	heart rate
fwRVLS	Free-wall right ventricular longitudinal strain
GLS	global longitudinal strain
GWI	global work index
GCW	global constructive work
GWW	global wasted work
GWE	global work efficiency
IVS	interventricular septum
K	blood potassium
LV EF	left ventricle ejection fraction
LAVi	left atrium volume index
MR	mitral regurgitation,
MW	myocardial work
NA	blood sodium
PACS	peak atrial contraction strain
PALS	peak atrial longitudinal strain
RVEDD	right ventricular end-diastolic diameter
RVFAC	right ventricular fractional area change
STE	speckle-tracking echocardiography
TAPSE	tricuspid annular plane systolic excursion
TDI-s	tissue Doppler imaging
SBP	systolic blood pressure
sPAP	systolic pulmonary artery pressure
TnHS	high-sensitivity troponin
